# How bad is the mere presence of a phone? A replication of Przybylski and Weinstein (2013) and an extension to creativity

**DOI:** 10.1371/journal.pone.0251451

**Published:** 2021-06-09

**Authors:** Claire Linares, Anne-Laure Sellier

**Affiliations:** Department of Marketing, HEC Paris, Jouy-en-Josas, France; Carnegie Mellon Univeristy, UNITED STATES

## Abstract

A 2013 article reported two experiments suggesting that the mere presence of a cellphone (vs. a notebook) can impair the relationship quality between strangers. The purpose of the present research is twofold: (1) closely replicate this article’s findings, and (2) examine whether there may be an impact of the mere presence of a phone on creativity, whether at a group- or an individual- level. In two experiments (*N* = 356 participants, 136 groups), we followed the original procedure in the 2013 article. In particular, groups of participants who had never seen each other before the study had a conversation in the mere presence of either a smartphone or a notebook. The participants then carried out creative tasks, in groups (Studies 1 and 2) or alone (Study 1). In both studies, we failed to replicate the original results on relationship quality. We also failed to find any effect of the mere presence of a phone on creativity. We discuss possible reasons which may have caused differences between our results and the original ones. Our main conclusion is an effect of the mere presence of a phone on relationship quality and creativity is at minimum harder to find than what was previously assumed in the literature. More generally, this research contributes to qualify the view that smartphones are harmful.

## Introduction

Through smartphones, people exchange in new ways, whether on social media or remote work applications. Most individuals are now connected to others almost constantly. To illustrate, American adults reportedly spend more than one hour each day networking on their smartphones [1] and more than 40% of English teenagers check social media after going to bed [2]. Even texting while sleeping—now referred to as sleep texting—keeps increasing among teenagers and college students [3]. Other than the extensive research documenting the heavy use of smartphones [e.g., 4–11], a few researchers published data suggesting that the mere presence of a mobile device is enough to harm social interactions [12, 13].

The possibility that the mere presence of a phone may actively and significantly shape the quality of social interactions is a formidably important question for society, given the ubiquity of mobile devices today [14, 15]. This research examines this question, and its purpose was twofold: first, we sought to replicate the findings that the mere presence of a phone can harm social interactions [12]. Second, we investigated whether similar harming effects of the mere presence of a phone may exist for creative cognition. In particular, we ask if groups and/or individuals engaged in a creative process perform differently, depending on whether a phone (vs. a notebook) is merely present.

### The mere presence of phones can harm social interactions: Evidence

Two articles present data suggesting a harmful effect of the mere presence of a phone on social interactions: Przybylski and Weinstein [12] and Misra, Cheng, Genevie, and Yuan [13]. In two studies collected in 2012 at the latest, Przybylski and Weinstein [12] shared concerning evidence that the mere presence of a mobile phone impairs relationship formation. In both studies, they found that dyads of strangers who were unobtrusively exposed to a cellphone felt a significantly lower sense of connection to their conversation partners, trusted them significantly less, and perceived less empathy from them than those exposed to a notebook.

In another study conducted in a coffee shop, Misra et al. [13] invited dyads of customers to have a conversation and noted whether the participants had a mobile device taken out during the conversation. In this study, the mobile device could be the participants’ own phones, tablets, or laptops. The findings were consistent with Przybylski and Weinstein [12]. Groups in presence of a mobile device reported a significantly lower connectedness to their partners and perceived empathy from them.

In the current research, we chose to focus on the relatively more controlled lab experiments in Przybylski and Weinstein [12]. In particular, they controlled for the type of mobile device (i.e., a phone) and included participants who did not know each other before the study. Given the formidable implications of their results [12], their reported effect of the mere presence of a mobile phone has been widely cited in academic research [e.g., 16–19] as well as in mainstream publications [e.g., 20–23]. Eight years after their article, however, it remains to be seen whether the adverse impact of the mere presence of a mobile phone on social interactions can be replicated, for at least two reasons. A first reason is that the evidence available remains limited, particularly in light of how widely cited this research is. We do note the interesting work by Courtright and Caplan [24], who concluded from a meta-analysis of only six studies that the mere presence of mobile phone effect is overall unsupported [12, 13, 25–27]. However, to the best of our knowledge, there is no direct evidence either confirming or invalidating the impact of the mere presence of a mobile phone on relationship formation, as Przybylski and Weinstein [12] documented it. Indeed, all replication attempts were only partial and substantially deviated from the original procedure. They involved either confederates, who had repeated conversations with participants, or pairs of participants who knew each other prior to the study [13, 25–27]. In addition, except in Misra et al.’s [13] field study, one of the conversation partners (either the confederate or one of the participants) was aware of the phone’s presence because of the procedure itself [25–27]. As a result, an attempt to replicate Przybylski and Weinstein’s [12] exact results would be valuable. A second reason for which a full replication of Przybylski and Weinstein [12] is helpful is that individuals may simply have gotten used to having smartphones to the point that there may be no mere phone presence effect *any longer*.

In this research, we provide an independent replication of Przybylski and Weinstein’s Study 1 [12]. This endeavor answers a call for direct replications before exploring an effect’s generalization [e.g., 28–30]. Such replications are particularly valuable for surprising phenomena exposed to an inflated risk of false-positive due to the publication of only successful experiments [31, 32]. In the case of Przybylski and Weinstein [12], another reason why a replication would be important is that their sample sizes were low (*N* = 37 groups for two conditions in Study 1; *N* = 34 groups for four conditions in Study 2) [29, 33]. Finally, as Przybylski and Weinstein’s [12] results were reported with covariates (gender, age, and positive affect), a non-robust effect is possible [29, 34].

### The mere presence of phones and creativity

The second aim of this research was to assess a possible impact of the mere and pervasive presence of a smartphone on our highest cognitive function: creativity [35]. Any creative effect of the mere presence of a phone—whether beneficial or detrimental—would be critical to document, considering the crucial role of creativity in society, to solve everyday problems [36, 37], as well as fuel artistic inspiration and innovation [38–41].

If there is a deleterious effect of the mere presence of a phone on the quality of social interactions [12], it is reasonable to consider that group creativity, in turn, may be compromised [e.g., 38, 42, 43]. Considerable past research gives hints of a possible negative effect of the mere presence of a phone on creativity. For example, extensive management research has highlighted the need for a supportive climate to enable team members to overcome their fear of judgment and voice their creative ideas [e.g., 42, 44–46]. Przybylski and Weinstein’s [12] findings would suggest that the mere presence of a phone may hurt group creativity, since it would presumably prevent an optimal relationship between group members. Another example is research showing that the mere presence of a mobile phone taxes working memory [47–51], which is another critical antecedent of creative thinking. Working memory is needed to transform preliminary mental idea components into creative ideas [52–55], and one type of working memory—short-term memory—is an additional aid in group-settings to remember others’ ideas and stimulate one’s idea generation, as well as to avoid idea loss when waiting to speak [56, 57].

Note that some research suggests that there may be a *positive* effect of the mere presence of a phone on creativity. The mere exposure to a mobile phone was shown to divert attention away from a focal task [47, 49–51]. In turn, research suggests that diverting the attention away from a creative task can be beneficial to generate ideas and solutions [40, 58–60]. Therefore, we tackled our exploratory investigation on group creativity with a two-tailed prediction, whereby the mere presence of a smartphone may enhance or impair group creative performance. We added a subsidiary exploration pertaining to individual-level creativity, as it could be affected by the mere presence of a smartphone through similar cognitive processes.

## Overview

**Fig 1:**
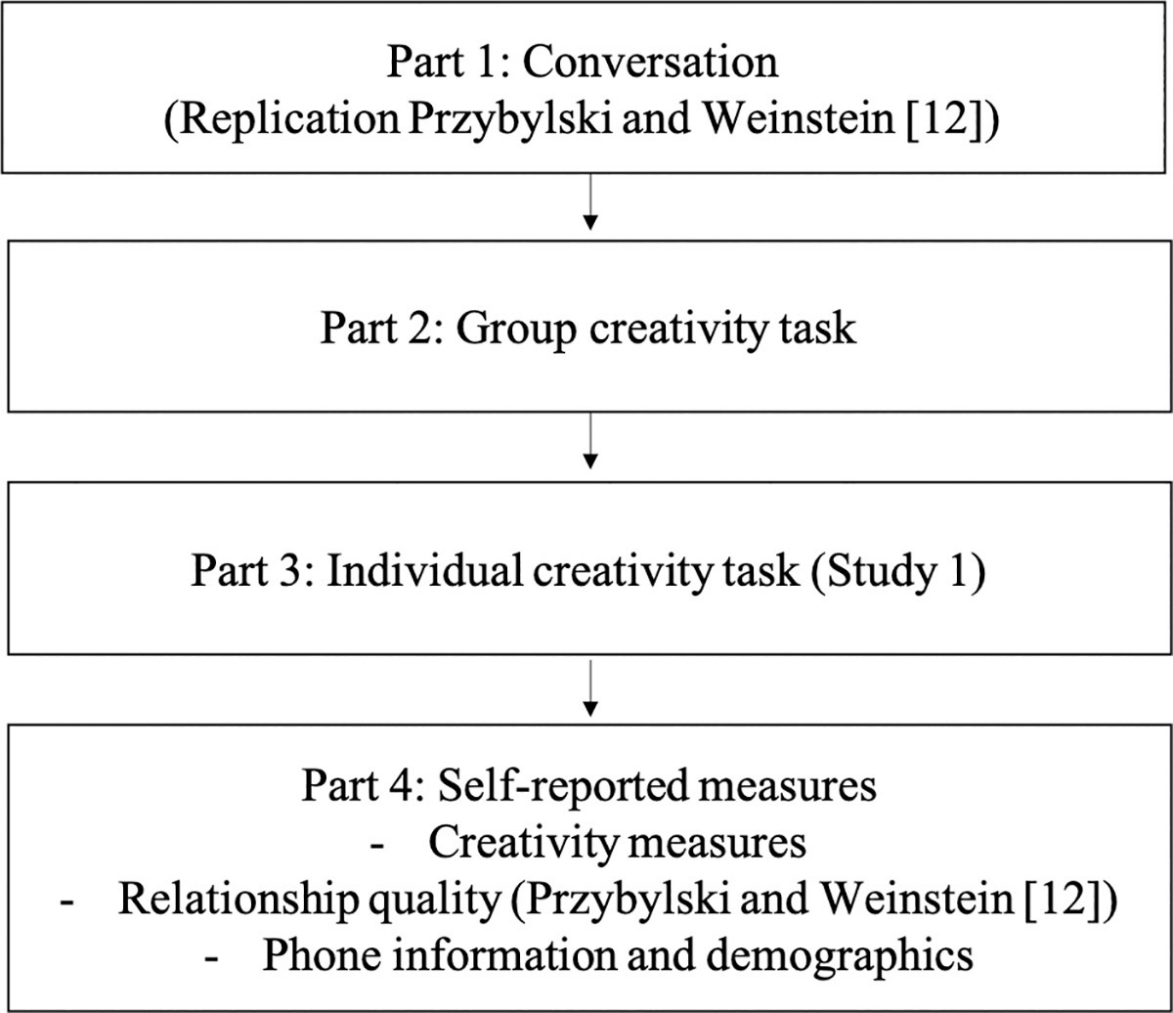
Procedure followed in Studies 1 and 2. The procedure was composed of several parts.

Our studies followed a two-step approach. We first followed a procedure as close as possible to Przybylski and Weinstein’s [12] Study 1 in order to replicate their findings. We then extended this procedure and added a group creativity task and an individual creativity task (Fig 1), in order to examine whether Przybylski and Weinstein’s [12] manipulation may rub off on creative cognition or the experience of the creative process.

In the first part, groups of participants were assigned to one of two conditions, the mere presence of either a smartphone or a notebook. Note that in our studies, we used a standard smartphone rather than a cellphone as in Przybylski and Weinstein [12], since it is today’s mainstream mobile device. Each group was instructed to have a conversation about an interesting event that occurred to them over the last month [12]. In the second part, participants were given a group creativity assignment where they had to create a toy for a child [37, 61], which was subsequently assessed by peer raters on originality and appropriateness [35, 37, 62]. As a last exploration on individual creativity (Study 1), participants did a remote associates test (RAT [63]). Following these tasks, participants answered a questionnaire with the social measures from Przybylski and Weinstein [12], as well as creativity measures. In Study 1, participants were split primarily in dyads, and occasionally in triads when one participant was absent. Because we found promising results on triads, we pursued our investigation in Study 2 with triads only.

## Methods

The studies were approved by the Institutional Review Board of the Centre de Recherche Multidisciplinaire Sorbonne Universités-INSEAD. All participants provided written consent. All the stimuli, additional pictures, R code, and datasets are accessible in the Supporting information. The data were collected in 2018, at a time where preregistration was not required for exploratory studies. For this reason, the two studies were not preregistered. We have deposited our laboratory protocols in protocols.io to ensure reproducibility of our results.

### Study 1

#### Participants

One hundred and forty-eight participants (62 men, 86 women, *M*_age_ = 22.82 years, *SD* = 3.24) from a European research lab were invited to complete a study about “Group Interactions” in return for 12 euros. Upon arriving at the lab, participants were randomly assigned to one of two conditions between-subjects (mere presence of a smartphone vs. mere presence of a notebook). Participants registered in sessions of four, initially to form two dyads per session. However, we had to compose triads when one participant in the session was absent. In total, 67 groups (53 dyads and 14 triads) completed the study successfully. One dyad was excluded from the analysis because one member had been speaking the local language for only four years, which could have prevented them from understanding all the instructions and prevented their group from fully understanding each other. In turn, we reasoned that it may have affected the relationship quality, and/or group and individual creativity. We kept participants who were not native speakers but had been speaking the local language for 15 years or more, allowing a good understanding of the instructions and the other group members. Note that including all participants in our analysis does not affect the results reported here. We report the results for the remaining 66 valid groups (52 dyads, 14 triads, 146 participants).

#### Procedure

**Fig 2:**
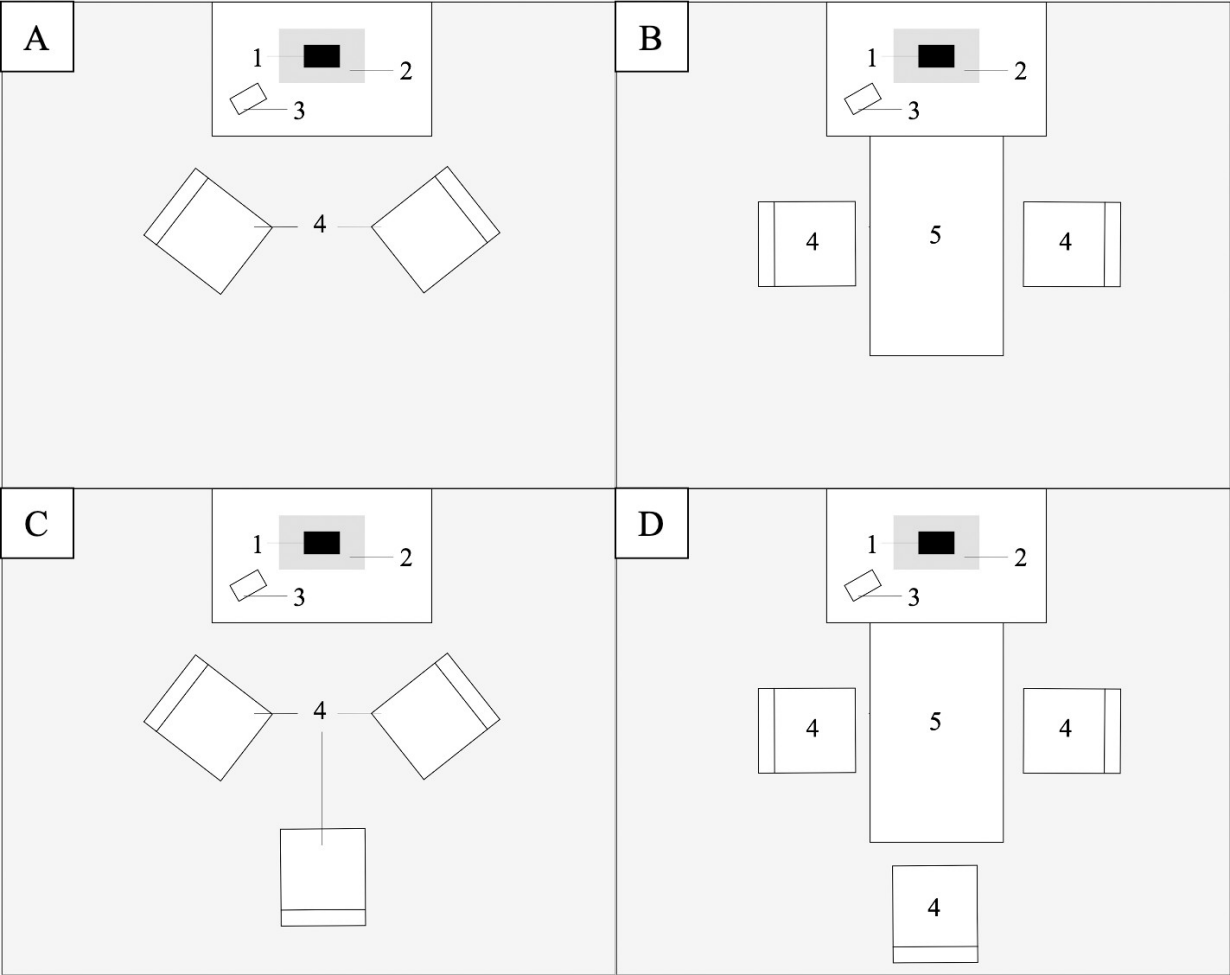
Layout of the study rooms during the conversation and creativity tasks (Studies 1 and 2). Schematic drawings of a bird’s eye view of the study rooms during the conversation for dyads (A) and triads (C), and during the creative tasks for dyads (B) and triads (D). In the rooms, the smartphone or notebook (1) was placed on a paper sleeve (2), next to a clock (3) on the main table. Chairs (4) were placed next to the main table. A second table (5) was added for the creative tasks. Adapted from Przybylski and Weinstein [12].

*Part 1: Replication of Przybylski and Weinstein [12].* At the beginning of the session, participants were asked whether they had ever talked to another member of the session before, including while waiting for the study to start. Based on their responses, we formed either one or two groups of participants, each of which was settled in a study room to start the study. Before entering the study room, participants left their personal belongings, including their phones, in a locker room, similarly to Przybylski and Weinstein [12]. Each group was assigned to its own room where there were two tables, the first one with the smartphone or notebook and the second one to be used for the creativity tasks. When the participants entered the room, chairs were placed next to the first table. For dyads, the layout of the room was strictly identical to the schema displayed in Przybylski and Weinstein [12] (Fig 2A), such that the smartphone or notebook was next to the participants but outside of their direct visual field. For triads, the layout was slightly adapted, such that one participant in the group faced the table while the two others were seated like the dyads (Fig 2C). The second table stayed in the corner of the room during the whole conversation task.

Przybylski and Weinstein [12] did not provide any information about the phone and notebook they used. We chose a nondescript black smartphone with a minimal design (S1 Picture) and a brand that was not well-known and not visible at first sight to avoid any brand effects, on creativity in particular [64]. In the absence of information, we positioned the smartphone’s screen facing up as has been done in other research on the mere presence of a smartphone [50]. In the control condition, the pocket notebook was chosen to be approximatively the same size and color (i.e., black) as the smartphone (S1 Picture). Underneath the object, we placed a purple paper sleeve instead of a book in Przybylski and Weinstein [12]. In both conditions, we chose to place a clock next to the smartphone or notebook to minimize the temptation for participants to look at the phone to monitor time (S1 Picture).

We emphasize that, as in the original article, we did not mention the smartphone or the notebook to participants during the tasks to focus our investigation on the effect of the smartphone’s mere presence. At the very end of the tasks and before moving to the final questionnaire, two participants asked whether someone had forgotten their phone and one participant in the notebook condition asked where it came from, that is whether it had been placed on the table during the tasks. As exploratory measures, we probed participants about the presence of the phone in the final questionnaire to see how many were aware of its presence and what were their thoughts, and again orally at the very end of the session both for the phone and the notebook. We report the results of these exploratory data for both Studies 1 and 2 in the appendix (S1 Appendix). Critically, note that the results we report below remain unchanged whether we remove (1) participants in the phone condition who were not aware of the presence of the phone, (2) groups where none of the participants was aware of the phone’s presence, and (3) groups where at least some members did not see the phone.

Once they were settled, participants were asked for their first names and were given the same relationship formation task as in Przybylski and Weinstein’s Study 1 [12]. In particular, they had to “discuss an interesting event that occurred to them over the past month” during 10 minutes [65].

*Part 2*: *Group creativity task*. When the time was up, we came back into the study room and added the second table next to the first one such that the smartphone or notebook remained in the same visual field as before (Fig 2B and 2D). We then gave participants the instructions for a widely used divergent creativity task, the “toy task” [e.g., 37, 61]. Each group was asked to design a new toy, as creative as possible, for a child from 5 to 11 to play with, using shapes from a given list (S2 Appendix). Participants had 15 minutes to draw the group’s toy on a piece of paper and were reminded of the time when there were only 5 minutes left. They also had to list five reasons why their toy was creative. Once the time was over, they wrote a small description about the toy and the way it worked.

*Part 3*: *Individual creativity task*. Next, participants were given an individual convergent creativity task, the remote associates test (RAT, [63, 66]; S3 Appendix). They had up to 10 minutes to solve 10 RATs while they were still in the study room in presence of the smartphone or notebook. They were instructed not to talk to each other.

*Part 4*: *Self-reported measures*. The final part of the study took place in individual cubicles where each participant completed three series of measures (S4 Appendix). First, participants indicated how creative they thought their group toy was on six items pertaining to originality and appropriateness (1–7 = “not at all” / “extremely creative / original / novel / useful / appropriate / practical”). A factor analysis of these items revealed that they loaded on two factors, originality and appropriateness, as in the bulk of prior creativity research [35]. For this reason, we averaged self-assessments of creativity, originality and novelty (α = 0.81) to create an originality score. We also averaged the usefulness, appropriateness and practicality items to create an appropriateness index (α = 0.67). Participants also indicated how satisfied they felt about the toy through three seven-point items that were averaged to form a satisfaction score (α = 0.87). Then, they completed creative process measures, all on seven-point scales. These measures captured the pleasantness (α = 0.86) and easiness of the process (*r* = 0.63, *p* < 0.001), the appreciation of group work (α = 0.83), group cohesion (α = 0.90), risk-taking (*r* = 0.73, *p* < 0.001), intrinsic motivation (*r* = 0.51, *p* < 0.001), feelings of competence, autonomy and control.

The second series of measures pertained to the initial conversation and were taken from Przybylski and Weinstein [12]. It included an assessment of relationship quality from the relatedness subscale of the Intrinsic Motivation Inventory [67]. As Przybylski and Weinstein [12], we used seven of the eight original items (α = 0.84). We will also report the results for the two items on trust separately as they are close to the trust measures reported in the original article (*r* = 0.46, *p* < 0.001; [12]). We added three new items on relationship quality (e.g., “I had the impression I could create a relationship with the other participant(s),” α = 0.73). Following Przybylski and Weinstein [12], we measured partner closeness with the Inclusion of Other in the Self Scale [65], partner empathy (“To what extent do you think your partner(s) accurately understood your thoughts and feelings about the topic”; Empathic Concern Scale, [68]) with an additional item on participants’ own empathy toward their partner(s). In addition, we measured interest in the discussion and self-reports of distraction. We included the PANAS scale to control for positive affect (α = 0.77, [69]) slightly deviating from Przybylski and Weinstein [12] who used the Emmons Mood Indicator [70]. The last part of the questionnaire included questions on phone ownership and usage, and ended with demographics questions (e.g., gender, age, language) and mood measures. Participants were probed for suspicions, both in the written questionnaire and orally. None of the participants guessed the purpose of the study. Finally, participants were debriefed and thanked.

#### Statistical analysis

As participants were nested into groups, our data structure is hierarchical, with the manipulation (smartphone vs. notebook) at level-2 and the dependent variables either at level-1 (e.g., relationship quality, RAT scores, self-reported measures) or at level-2 (group toy creativity scores), requiring different analytical strategies (Table 1).

**Table 1:** Summary of main analyses for the different categories of dependent variables (Studies 1 and 2).

Category of dependent variables	Main analysis
Relationship measures (Przybylski and Weinstein [12])	Multilevel analysis: random intercept model with mere presence of smartphone as level-2 fixed effect, and gender, age, and positive affect covariates as level-1 fixed effects
Creativity scores of group toys rated by peer judges	*t* test: mere presence of smartphone as predictor (no covariates)
Remote associates test (Study 1)	Multilevel analysis: random intercept model with mere presence of smartphone as level-2 fixed effect (no covariates)
Self-assessments of creativity	Multilevel analysis: random intercept model with mere presence of smartphone as level-2 fixed effect (no covariates)
Creative measures	Multilevel analysis: random intercept model with mere presence of smartphone as level-2 fixed effect (no covariates)

For level-1 dependent variables, the standard analysis for hierarchical data is multilevel linear modelling, which Przybylski and Weinstein [12] used. We conducted identical analyses for our replication measures. The model is specified as follows:

$$Y_{ij}=\left(b_{0}+u_{0j}\right)+b_{1}X_{ij}+b_{2}Gender_{ij}+b_{3}Age_{ij}+b_{4}\mathrm{Positive}\;affect_{ij}+\varepsilon_{ij}$$ (1)

where Y_*ij*_ is the dependent variable (e.g., relationship quality) of an individual *i* of group *j; b*_0_ and *u*_0*j*_ are the fixed and random intercept coefficients; *b*_1_ is the parameter associated to the fixed effect of the independent variable X_*ij*_ (smartphone vs. notebook); *b*_2,_ *b*_3,_ and *b*_4_ are the parameters associated to the covariates (gender, age, and positive affect). The model is thus a random intercept one where only the intercept can vary across groups. We used a maximum likelihood estimation method which provides more accurate estimates of the fixed effect parameters [71] as we were primarily interested in the fixed effect of the smartphone versus notebook. The age and positive affect covariates were grand-mean centered. The positive affect variable was the average of the 10 positive affect items from the PANAS scale [69]. The conclusions are the same when removing all covariates from the model. It should be noted that some of the intraclass correlations were low (below 0.001), which indicates a limited impact of clustering on the data [72]. For the level-1 dependent variables other than relationship quality measures (i.e., RAT scores, other self-reported measures), we also used a random intercept multilevel model but we did not include the covariates in our main analyses. For level-2 toy creativity scores, we conducted *t* tests as both the manipulation and the dependent variables were at the group-level.

In Study 1, we first conducted analyses on dyads to closely follow Przybylski and Weinstein [12]. We then extended the analyses to triads, and finally to dyads and triads taken together. Study 2 included only triads. The level of significance was set at a two-tailed *p* value of 0.05. Multilevel analyses were performed in R using the *nlme* package for the model estimations and the *performance* package for the ICCs. Results remain the same with SPSS and the R package *lme4*. *T* tests were performed in SPSS.

#### Results

*Relationship quality (Przybylski and Weinstein [12]).* Tables 2 and 3 present the results of our multilevel analyses for dyads compared with results reported in Przybylski and Weinstein [12]. In contrast to Przybylski and Weinstein [12], we did not find a significant effect of the mere presence of a mobile phone (vs. notebook) on any of the original relationship measures, all *p*s > 0.45. We also did not find any effect on new measures of relationship quality, all *p*s > 0.18. As expected, positive affect was significantly and positively related to most of the relationship measures (all *b*s > 0, *p*s < 0.01) except to the trust items (*p* = 0.88), while age and gender were not related to any measures (all *p*s > 0.14). Subsequent analyses on triads only, and on dyads and triads taken together, revealed no effect of the mere presence of a smartphone on relationship measures (S1 Table).

**Table 2:** Descriptive statistics of relationship measures for dyads (Study 1). Means and standard deviations by condition, sample sizes. Comparison with information reported in Przybylski and Weinstein [12].

		Phone	Notebook
	*N*_groups_	*M (SD)*	*M (SD)*
**Przybylski and Weinstein [12]. Study 1**			
** Relationship quality**	37	2.78 (0.79)	
** Partner closeness**	37	3.57 (1.46)	
**Przybylski and Weinstein [12]. Study 2**			
** Relationship quality**	34	4.98 (1.09)	
** Trust**	34	3.25 (1.01)	
** Partner empathy**	34	4.98 (1.09)	
**Study 1 (this paper)—Dyads only**			
** Relationship quality**	52	5.26 (1.02)	5.09 (1.02)
** Trust (subscale)**	52	5.74 (0.98)	5.76 (1.10)
** Additional items relationship quality**	52	5.28 (1.18)	5.08 (1.30)
** Partner closeness**	52	3.92 (1.61)	3.83 (1.56)
** Partner empathy**	52	3.85 (0.83)	3.94 (0.85)
** Own empathy**	52	3.88 (0.83)	3.65 (1.03)
** Experience appreciation**	52	5.71 (1.23)	5.78 (1.05)
** Concentration**	52	5.99 (1.07)	6.02 (1.01)

Descriptive statistics for triads, and dyads and triads together are reported in the S1 Table.

**Table 3:** Multilevel analyses for relationship measures of dyads (Study 1). Intraclass correlations, fixed effects estimates, and significance tests for the manipulation and covariates. Comparison with results reported in Przybylski and Weinstein [12].

		Fixed effect level-2 phone			Fixed effect level-1 covariate gender			Fixed effect level-1 covariate age^a^			Fixed effect level-1 covariate positive affect^a^		
* *	ICC	*b*	*t*	*p*	*b*	*t*	*p*	*b*	*t*	*p*	*b*	*t*	*p*
**Przybylski and Weinstein [12]. Study 1**													
** Relationship quality**	0.43–0.51	-0.99	3.08	0.004	-20	-0.61	0.55	-0.04	-0.03	0.12	0.80	2.99	< 0.001
** Partner closeness**	0.43–0.51	-0.39	-2.56	0.02	-0.00- -0.09	-0.06- -0.64	> 0.51	-0.00- -0.09	-0.06- -0.64	> 0.51	-0.00- -0.09	-0.06- -0.64	> 0.51
**Przybylski and Weinstein [12]. Study 2**													
** Relationship quality**	0.39–0.47	-0.19	-2.29	0.03	0.01–0.09	0.13–1.28	> 0.20	0.01–0.09	0.13–1.28	> 0.20	0.01–0.09	0.13–1.28	> 0.20
** Trust**	0.39–0.47	-0.36	-3.76	< 0.001	0.02–0.13	-0.13–1.50	> 0.12	0.02–0.13	-0.13–1.50	> 0.12	0.02–0.13	-0.13–1.50	> 0.12
** Partner empathy**	0.39–0.47	-0.37	-3.60	0.002	-0.08	-0.29	0.77	0.04	1.92	0.06	0.21	1.55	0.13
**Study 1 (this paper)—Dyads only**													
** Relationship quality**	0.10	0.16	0.75	0.456	-0.20	-1.01	0.318	0.02	0.59	0.557	0.50	2.85	0.006
** Trust (subscale)**	< 0.001	-0.03	-0.15	0.883	-0.10	-0.49	0.627	0.04	1.30	0.199	-0.03	-0.15	0.881
** Additional items relationship quality**	< 0.001	0.19	0.83	0.413	-0.12	-0.50	0.623	0.01	0.32	0.752	0.79	3.74	< 0.001
** Partner closeness**	< 0.001	0.08	0.29	0.772	-0.44	-1.44	0.156	-0.05	-1.14	0.259	0.99	3.73	< 0.001
** Partner empathy**	< 0.001	-0.11	-0.69	0.495	-0.23	-1.44	0.157	-0.01	-0.32	0.748	0.52	3.71	0.001
** Own empathy**	< 0.001	0.23	1.33	0.190	0.01	0.04	0.971	-0.01	-0.49	0.627	0.60	3.80	< 0.001
** Experience appreciation**	< 0.001	-0.07	-0.37	0.714	-0.07	-0.34	0.733	-0.00	-0.08	0.933	0.96	5.29	< 0.001
** Concentration**	< 0.001	-0.05	-0.25	0.805	-0.29	-1.48	0.146	0.03	0.98	0.332	0.55	3.13	0.003

Results for triads, and dyads and triads together, as well as detailed confidence intervals are reported in the S1 Table.

^a^Age and positive affect are grand-mean centered.

*Group creativity*. To assess the creativity of the toys created by groups of participants, 13 peer judges were invited to the lab to independently rate each toy on a series of measures in exchange for 25€. The judges were invited in sessions of one or two and brought to a room where all the toy drawings, descriptions and reasons why they were creative, were displayed on the walls. All judges were blind to the hypotheses and conditions, and the toys were presented in one of three random orders. After reading the instructions of the toy task and taking an overview of all the toys, they were first asked to rate each one on its overall creativity. Then, in line with previous research, they had to indicate their ratings on sub-dimensions of creativity, pertaining to originality (i.e., originality, novelty) and appropriateness (i.e., usefulness, practicality, appropriateness) [39, 41]. All the criteria were evaluated on ten-point scales (e.g., 1 = “not at all creative” to 10 = “extremely creative”). Finally, the judges had to indicate how many euros they thought parents would be willing to pay for each toy and completed a final questionnaire including demographics.

We first averaged the 13 valid judges’ ratings on each item to obtain mean judges’ creativity (α = 0.85), originality (α = 0.84), and novelty (α = 0.85) scores for each toy. These three scores loaded on one factor with high reliability (α = 0.96) and were thus averaged to create an overall originality index. Similarly, we computed mean judges’ usefulness (α = 0.61) and appropriateness scores (α = 0.65), and then averaged them to obtain an overall appropriateness index (*r* = 0.69, *p* < 0.001). We analyzed the results of each judge’s practicality ratings separately because the interrater reliability for this item was low (α = 0.37). We created an index for the estimated parents’ willingness to pay by averaging all judges’ amounts (α = 0.88), which we then logtransformed as it was not normally distributed (Kolmogorov-Smirnov test, *D*(66) = 0.20, *p* < 0.001).

Overall, we found good levels of average originality (*M* = 4.75, *SD* = 1.34, dyads and triads) and appropriateness (*M* = 5.79, *SD* = 0.83). We conducted a series of *t* tests on dyads which did not reveal any effect of the mere presence of a smartphone versus a notebook on the overall originality, appropriateness, and logtransformed willingness to pay indices, all *p*s > 0.41, or on any of the judges’ practicality ratings (for one judge, *t*(50) = 1.46, *p* = 0.15; for the other judges, all *p*s > 0.27; S2 Table). However, on triads, *t* tests revealed a significant effect of the smartphone’s mere presence versus the notebook’s on the appropriateness of the toys, *t*(12) = -3.03, *p* = 0.01, 95% CI [-1.57, -0.26], *d* = 1.69 (S2 Table), such that the toys created in presence of the smartphone were judged as *more* appropriate (*M*_*smart*phone_ = 6.53, *SD*_*smart*phone_ = 0.61) than those created in presence of the notebook (*M*_notebook_ = 5.62, *SD*_notebook_ = 0.36).

For triads, there was also a significant effect on the willingness to pay, *t*(12) = -2.79, *p* = 0.02, 95% CI [-0.51, -0.06], *d* = 1.55, such that judges estimated a higher parents’ willingness to pay for toys created in the mere presence of a smartphone (*M*_*smart*phone_ = 1.43, *SD*_*smart*phone_ = 0.22, logtranformed values) than of a notebook (*M*_notebook_ = 1.14, *SD*_notebook_ = 0.09). The toys created in the presence of the smartphone were also judged as marginally more original (*M*_*smart*phone_ = 5.84, *SD*_*smart*phone_ = 1.38; *M*_notebook_ = 4.57, *SD*_notebook_ = 0.94*; t*(12) = -1.82, *p* = 0.09, 95% CI [-2.79, 0.25], *d* = 1.02). When analyzing dyads and triads together, there were only marginal effects of the mere presence of the smartphone on the toy creativity item, *p* = 0.05, and estimated parents’ willingness to pay, *p* = 0.07, in the same direction as triads, and no significant effect on any of the other creativity scores.

*Individual creativity*. The performance at the remote associates test (RAT) was computed with two different methods. The first one used a predetermined list of correct answers (e.g., “Monarchy” associated with the given words “King”, “England” and “Crown”). The second one allowed for some flexibility in the answers (e.g., “Queen” was accepted in the previous example). However, according to multilevel analyses, no effect for participants in dyads was observed on either RAT score (*M*_correct_ = 2.73, *SD* = 1.35; *M*_flexible_ = 4.24, *SD* = 1.21, dyads and triads), both *p*s > 0.70 (S3 Table). There was also no difference in the number of RAT answers provided, *t*(50) = 0.76, *p* = 0.45 *(M* = 8.32, *SD* = 1.69, dyads and triads). Results are the same for triads, and dyads and triads combined (S3 Table).

*Self-assessments of toy creativity*. Multilevel analyses did not reveal any significant effect of the mere presence of a smartphone on dyads’ self-assessed toy originality, *t*(50) = -0.07, *p* = 0.95; self-assessed toy appropriateness, *t*(50) = -1.12, *p* = 0.27; or satisfaction with the toys, *t*(50) = -0.12, *p* = 0.91. Conclusions remain the same for triads, and dyads and triads combined (S3 Table), except for a marginal effect of the smartphone on triads’ satisfaction with their toys such that triads in the phone condition were less satisfied than those in the notebook condition, *t*(12) = -1.79, *p* = 0.10.

*Creativity process measures*. We ran multilevel analyses on all the toy creative process measures (S3 Table). We found no difference between the smartphone and notebook conditions on ease or enjoyment of the creative process, group cohesion, and group work during the toy creativity task, all *p*s > 0.34. Furthermore, we did not find any effects on risk-taking, motivation, feelings of competence, power, control (all *p*s > 0.21), except a marginal effect on autonomy (*t*(50) = 1.89, *p* = 0.07), such that participants in presence of the smartphone felt more autonomous. Results remain the same for triads alone, and dyads and triads together, with the exception of a significant effect of the smartphone’s presence on feeling of autonomy, *b* = 0.42, *t*(64) = 2.04, *p* = 0.05, 95% CI [0.01, 0.82], such that groups in presence of the smartphone (vs. the notebook) felt more autonomous when they were creating the toy.

#### Discussion

In Study 1, we failed to replicate Przybylski and Weinstein’s [12] finding that the mere presence of a smartphone impairs relationship quality during a conversation between strangers. We also did not find any effect of the device’s presence on creativity, except for triads. Specifically, toys created by triads in the mere presence of a smartphone (vs. a notebook) were judged as more appropriate, and marginally more original. Judges also thought parents would be willing to pay more for them. These findings are consistent with research suggesting that groups can be more or less creative depending on the number of members [e.g., 73–75]. In Study 2, we sought to confirm these results by conducting a study with triads only.

### Study 2

Study 2 aimed to replicate the preliminary findings of Study 1 and confirm the effects of the mere presence of a smartphone on creativity with a greater number of triads. In this study, we also attempted to replicate Przybylski and Weinstein’s [12] finding a second time. Overall, the procedure of this study was similar to that of Study 1.

#### Participants

Two hundred and thirteen participants (76 men, 137 women, *M*_age_ = 21.89 years, *SD* = 3.08) from a European research lab took part in the study in return for 10 euros. They were split in 71 triads randomly assigned to one of two conditions between-subjects (mere presence of a smartphone vs. of a notebook). We excluded one triad in which one member had been speaking the local language for only five years. All other participants included in the analysis had been speaking the local language for 10 years or more. The results hold when including all participants who took the study. This left 70 valid triads (210 participants).

#### Procedure

This study followed the same procedure as Study 1, with the following exceptions. After going through the discussion and toy tasks, participants directly completed the final questionnaire without taking the individual RAT task. During the tasks, two participants in the phone condition and one participant in the notebook condition asked whether they were recorded, without mentioning the smartphone or notebook. No participants made spontaneous comments about the presence of the objects. The final questionnaire included a slightly different set of measures (S5 Appendix). In this study, the self-assessment of toy creativity was measured on creativity, novelty, originality, innovativeness, usefulness, and appropriateness on seven-point scales (e.g., 1 = “not at all creative” to 7 = “extremely creative”). Self-assessments of toy creativity, novelty, originality and innovativeness loaded on one factor (α = 0.84) and were thus averaged to form a self-assessed originality index, and usefulness and appropriateness were averaged to form a self-assessed appropriateness index (*r* = 0.40, *p* < 0.001). In addition, we measured ease (*r* = 0.50, *p* < 0.001) and enjoyment of the creative process (α = 0.85), group cohesion (α = 0.91), risk-taking (*r* = 0.61, *p* < 0.001), motivation (*r* = 0.54, *p* < 0.001), and feelings of competence and autonomy. We also included items about feelings of busyness, mind wandering [58] and approach/avoidance [41, 76]. As relationship measures, we only kept the Intrinsic Motivation Inventory scale (α = 0.83, [67]) used in both studies in Przybylski and Weinstein [12], including the two items measuring trust (*r* = 0.53, *p* < 0.001). We did not include other measures from the original article, but we had three other items on relationship quality (α = 0.78) and two items on the appreciation of and interest in the conversation (*r* = 0.68, *p* < 0.001).

#### Results

In Study 2, we followed the same analytical methods as in Study 1.

*Relationship quality (Przybylski and Weinstein [12]).* Tables 4 and 5 present the detailed results of the multilevel analyses controlling for gender, age and positive affect (α = 0.84, PANAS [69]). As in Study 1, there was no significant difference between both conditions on relationship quality (including trust), and additional relationship items, all *p*s > 0.29. Also, no difference was observed on experience appreciation and interest, *t*(68) = -0.74, *p* = 0.47. Gender was related to relationship quality and experience appreciation, such that women indicated a higher relationship quality and appreciation of the conversation, both *p*s < 0.03. Positive affect was positively related to all relationship measures, all *p*s < 0.001.

**Table 4:** Descriptive statistics of relationship measures (Study 2). Means and standard deviations by condition, sample sizes. Comparison with information reported in Przybylski and Weinstein [12].

		Phone	Notebook
	*N*_groups_	*M (SD)*	*M (SD)*
**Przybylski and Weinstein [12]. Study 1**			
** Relationship quality**	37	2.78 (0.79)	
** Partner closeness**	37	3.57 (1.46)	
**Przybylski and Weinstein [12]. Study 2**			
** Relationship quality**	34	4.98 (1.09)	
** Trust**	34	3.25 (1.01)	
** Partner empathy**	34	4.98 (1.09)	
**Study 2 (this paper)—Triads**			
** Relationship quality**	70	4.96 (1.06)	4.91 (1.11)
** Trust (subscale)**	70	5.65 (1.28)	5.62 (1.23)
** Additional items relationship quality**	70	5.32 (1.13)	5.17 (1.39)
** Experience appreciation**	70	5.67 (1.24)	5.84 (1.21)

**Table 5:** Multilevel analyses for relationship measures (Study 2). Intraclass correlations, fixed effects estimates, and significance tests for the manipulation and covariates. Comparison with results reported in Przybylski and Weinstein [12].

		Fixed effect level-2			Fixed effect level-1			Fixed effect level-1			Fixed effect level-1		
		phone			covariate gender			covariate age^a^			covariate positive affect^a^		
	ICC	*b*	*t*	*p*	*b*	*t*	*p*	*b*	*t*	*p*	*b*	*t*	*p*
**Przybylski and Weinstein [12]. Study 1**													
** Relationship quality**	0.43–0.51	-0.99	3.08	0.004	-20	-0.61	0.55	-0.04	-0.03	0.12	0.80	2.99	< 0.001
** Partner closeness**	0.43–0.51	-0.39	-2.56	0.02	-0.00-	-0.06- -0.64	> 0.51	-0.00-	-0.06- -0.64	> 0.51	-0.00-	-0.06- -0.64	> 0.51
											-0.09		
					-0.09			-0.09					
**Przybylski and Weinstein [12]. Study 2**													
** Relationship quality**	0.39–0.47	-0.19	-2.29	0.03	0.01–0.09	0.13–1.28	> 0.20	0.01–0.09	0.13–1.28	> 0.20	0.01–0.09	0.13–1.28	> 0.20
** Trust**	0.39–0.47	-0.36	-3.76	< 0.001	0.02–0.13	-0.13–1.50	> 0.12	0.02–0.13	-0.13–1.50	> 0.12	0.02–0.13	-0.13–1.50	> 0.12
** Partner empathy**	0.39–0.47	-0.37	-3.60	0.002	-0.08	-0.29	0.77	0.04	1.92	0.06	0.21	1.55	0.13
**Study 2 (this paper)—Triads**													
** Relationship quality**	0.10	0.10	0.69	0.494	0.40	2.70	0.008	0.00	0.03	0.980	0.74	6.77	< 0.001
** Trust (subscale)**	0.10	0.05	0.27	0.788	0.20	1.09	0.278	0.00	0.15	0.880	0.52	3.78	< 0.001
** Additional items**	0.12	0.20	1.06	0.295	0.33	1.94	0.054	-0.00	-0.15	0.881	0.98	8.06	< 0.001
**relationship quality**													
** Experience appreciation**	0.14	-0.12	-0.74	0.465	0.38	2.24	0.026	-0.00	-0.05	0.961	0.72	5.71	< 0.001

Confidence intervals are reported in the S4 Table.

^a^Age and positive affect are grand-mean centered.

*Group creativity*. *N* = 12 students from a European business school assessed the originality and appropriateness of the toys created by groups in exchange for 25 euros. They were invited in sessions of two to six participants in a large room where all the toy designs were displayed in one of three random orders. They were given the same toy task instructions as in Study 1 and proceeded to rate each design in terms of overall creativity (1–10 = “not at all / extremely creative”), originality (3 items, 1–10, “not at all” / “extremely original / novel / innovative”) and appropriateness (2 items, 1–10, “not at all” / “extremely useful / appropriate”). Finally, they evaluated how much parents would be willing to pay for each toy and completed a final questionnaire including demographics. We first averaged the 12 valid judges’ ratings on overall creativity (α = 0.86), originality (α = 0.88), novelty (α = 0.86), and innovativeness (α = 0.88). These four scores loaded on a single factor (α = 0.98) and were thus averaged to obtain an overall originality index (*M* = 4.92, *SD* = 1.46). Then, we averaged the 12 judges’ ratings on usefulness (α = 0.75) and appropriateness (α = 0.62), and computed their average (*r* = 0.70, *p* < 0.001) to create an overall appropriateness index (*M* = 5.86, *SD* = 0.92). The 12 raters’ estimated parents’ willingness to pay had a high Cronbach alpha (α = 0.87) and were averaged to create a willingness to pay index, that we logtransformed (Kolmogorov-Smirnov test, *D*(70) = 0.24, *p* < 0.001).

A series of *t* tests did not reveal any significant differences between conditions on overall originality, *t*(68) = 0.16, *p* = 0.88, overall appropriateness, *t*(68) = 1.19, *p* = 0.24, or willingness to pay, *t*(68) = 0.79, *p* = 0.43 (S5 Table).

*Self-assessments of toy creativity*. Multilevel analyses did not reveal any effect of the mere presence of a smartphone on self-assessed toy originality, self-assessed toy appropriateness, or satisfaction, all *p*s > 0.88 (S6 Table).

*Creativity process measures*. We ran multilevel analyses on all process measures (S6 Table) and no difference emerged between conditions on easiness or enjoyment of the creative process, group cohesion, risk-taking or motivation, competence or autonomy, all *p*s > 0.20. There was no effect on mind wandering, feelings of busyness, and approach/avoidance measures either, all *p*s > 0.13.

#### Discussion

In contrast to Przybylski and Weinstein’s [12] results and as in Study 1, Study 2 failed to reveal any effect of the mere presence of a smartphone [vs. a notebook) on relationship quality following a conversation between strangers. In addition, we failed to replicate the results observed in Study 1 on triads’ creativity.

## General discussion

In two studies, we failed to replicate Przybylski and Weinstein’s [12] results showing an adverse effect of the mere presence of a mobile phone on relationship formation, when considering both dyads and triads of strangers. We also did not find any effect of the mere presence of a mobile phone on any aspect of creative cognition, examining both divergent and convergent creativity processes and outputs, and both group and individual creativity. These results suggest that mere presence of a mobile phone may not be as harmful as has been previously claimed [12, 13].

### Non-replication of Przybylski and Weinstein (2013)

The failed replication result directly adds to the line of research on the negative consequences of the mere presence of a mobile device [12, 13, 49–51], by suggesting that this negative influence may not be as marked as was previously assumed. If we cannot exclude that there might be other instances where this presence is harmful, our findings at least point out the fragility of the phenomenon. At a broader level, our results also nuance the dominant view that smartphones and technology are harmful, adding to burgeoning research that is casting doubt on the pervasiveness of their negative effects [e.g., 4, 8, 77]. Our findings also complement research on mere exposure effects [78–80]. Finally, our research supports the importance of replications [e.g., 29, 81, 82]: Until an effect has been independently replicated, researchers need to remain cautious in assuming its existence [28, 31].

Despite our best efforts to conduct the closest replication as possible, our studies contain limitations. First, null effects do not invalidate an effect. It may be that the mere presence of the phone is harmful in different populations from those we sampled (e.g., in populations where the use of the smartphone may not be as pervasive as in large European cities). Second, even though our sample sizes were much greater than those in Przybylski and Weinstein [12], they may not have provided enough power to detect an effect [77]. Third, we measured relationship quality after the creative tasks and self-reports on the creative process, which might have wiped out the effect due to fatigue or contamination.

Is there a way to reconcile our results with those of Przybylski and Weinstein [12]? We see several possibilities, all of which relate to the timing of our experiments. We collected our data in 2018, compared to Przybylski and Weinstein (in or before 2012) [12]. The first possibility is that people might have simply gotten used to the presence of mobile devices, which could make them immune to their mere presence. Relatedly, our sample included mainly participants in their twenties, that belong to a generation who grew up with smartphones, and thus who might find their presence very natural. Another reason could be that the technology has evolved greatly between 2012 and 2018. Przybylski and Weinstein [12] used a cellphone, while we used a smartphone. Indeed, the number of smartphone users worldwide has more than doubled during this interval [83]. Additionally, people have developed strong bonds to them, although this is less likely to explain our results since in our studies, participants were exposed to a lab device [77, 84, 85]. If any of these conjectures were true, then our findings would provide an updated assessment of the social consequences of the mere presence of a mobile phone, suggesting that its effect was short-lived.

### Null effect on creativity

The absence of evidence supporting a link between the mere presence of a smartphone and creativity advances the exploration of the effects of technology on idea generation. Our results suggest that the mere presence of a technological device like a smartphone may not affect general measures of creativity, whether positively or negatively. Previous research has pointed out the critical role of the environment for creativity which can be affected by incidental cues like sound or background color or the presence of physical objects [66, 86–90]. Relatedly, a lay belief exists that a creative environment should be free from any distractions [91, 92]. Our findings suggest that creativity may not be that sensitive to the mere presence of technology.

One limitation of this investigation is that participants did the creativity tasks only after the conversation. However, in the real-world, creativity endeavors are rarely isolated from other processes, and we would argue that this improbably caused our null effects.

### Implications and avenues for future research

The main implication of this research is to moderate calls to completely isolate from smartphones’ presence. It may not be necessary to ban even switched off smartphones from the dinner table or to enforce strict cellphone policies in organizations [93–96]. For instance, the American, French and British governments have a no-phone policy during meetings, whereby each member has to leave their phones at the entry of the meeting room [97]. Our findings suggest that these constraints could be partly released, at least for meetings in which there is no concern that sensible information may be recorded.

Of course, when people’s own smartphones are present, the temptation to use them might still be detrimental for social interactions or creativity [19, 98, 99]. In Misra et al. [13] for instance, acquaintances in the mere presence of their own mobile devices experienced a lower relationship quality, although similarly to Przybylski and Weinstein [12], this finding remains to be replicated. In a study we do not report here, we investigated whether individual idea generation could be affected by the presence of one’s own smartphone. Again, we observed no effect on creative cognition. At this point, it is our view that there is not much influence of the mere presence of a phone on creative cognition. What may be worthwhile investigating are ways in which smartphones may impact creativity, other than via their mere presence. For instance, future research could explore the difference between generating ideas on one’s smartphone rather than on one’s personal computer [84, 85]. This could also be interesting when exchanging ideas with other people, in the wake of research on electronic brainstorming [100–102]. More broadly, the topic of creativity and technology still offers a wide field of investigation.

## Conclusion

We did not replicate Przybylski and Weinstein’s [12] finding that the mere presence of a mobile device impairs relationship quality, nor did we find any effect of this presence on creativity. There is one practical recommendation arising from our results: next time you meet a stranger or work on a creative task, you may leave your phone on the table. Just turn on the *airplane mode*.

## Supporting information

S1 Picture Pictures of the smartphone and notebook in the study room (Studies 1 and 2). (DOCX)

S1 Appendix Additional information regarding the manipulation and participants’ reactions to the presence of the smartphone and the notebook (Studies 1 and 2). (DOCX)

S2 Appendix Instructions for the toy creativity task (Studies 1 and 2). Translation from local language.
(DOCX)

S3 Appendix Remote associates test (Study 1). Translation from local language.
(DOCX)

S4 Appendix Final questionnaire (Study 1). Translation from local language.
(DOCX)

S5 Appendix Final questionnaire (Study 2). Translation from local language.
(DOCX)

S1 Table Multilevel analyses for relationship measures of all group sizes (Study 1). Means and standard deviations by condition, sample sizes. Intraclass correlations, fixed effects estimates, significance tests, and confidence intervals for the manipulation and covariates. Comparison with results reported in Przybylski and Weinstein [12].
(XLSX)

S2 Table *t* tests for toy creativity scores of all group sizes (Study 1). Means and standard deviations by condition, *t* test values, significance tests, and confidence intervals.
(XLSX)

S3 Table Multilevel analyses for self-assessments of creativity and creative measures of all group sizes (Study 1). Means and standard deviations by condition, intraclass correlations, fixed effect estimates, significance tests, and confidence intervals.
(XLSX)

S4 Table Multilevel analyses for relationship measures with confidence intervals (Study 2). Means and standard deviations by condition, sample sizes. Intraclass correlations, fixed effects estimates, significance tests, and confidence intervals for the manipulation and covariates. Comparison with results reported in Przybylski and Weinstein [12].
(XLSX)

S5 Table *t* tests for toy creativity scores (Study 2). Means and standard deviations by condition, *t* test values, significance tests, and confidence intervals.
(XLSX)

S6 Table Multilevel analyses for self-assessments of creativity and creative measures (Study 2). Means and standard deviations by condition, intraclass correlations, fixed effect estimates, significance tests, and confidence intervals.
(XLSX)

S1 Code R code for multilevel analyses and ICCs (Studies 1 and 2). (R)

S1 Dataset Self-reported measures (Study 1). Relationship measures, self-assessment of creativity, and creative measures.
(XLSX)

S2 Dataset Creativity scores for group toys (Study 1). Ratings by peer judges.
(XLSX)

S3 Dataset Self-reported measures (Study 2). Relationship measures, self-assessment of creativity, and creative measures.
(XLSX)

S4 Dataset Creativity scores for group toys (Study 2). Ratings by peer judges.
(XLSX)

S1 File Variable information (Studies 1 and 2). Variable names, labels, and values for all datasets.
(XLSX)
