# Supplementary material for: How bad is the mere presence of a phone? A replication of Przybylski and Weinstein (2013) and an extension to creativity
Source: PLoS One. 2021 Jun 9;16(6):e0251451. doi: 10.1371/journal.pone.0251451 (PMC8189469; doi:10.1371/journal.pone.0251451)

**S1 Picture. Pictures of the smartphone and notebook in the study room (Studies 1 and 2).**

1. Smartphone versus notebook (Study 1)

**
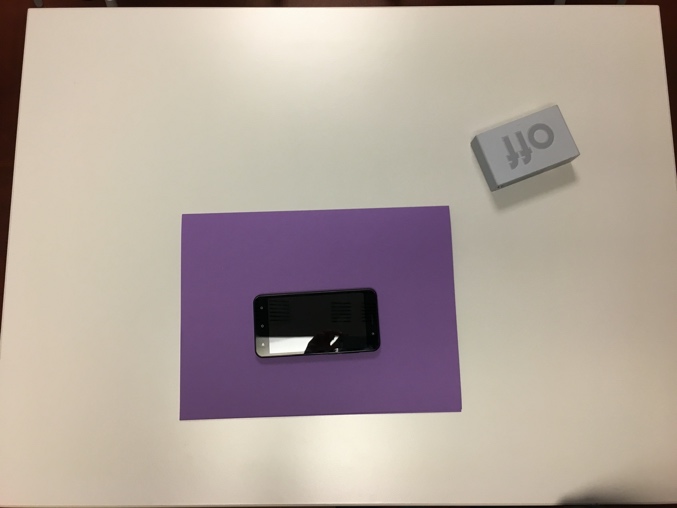
** **
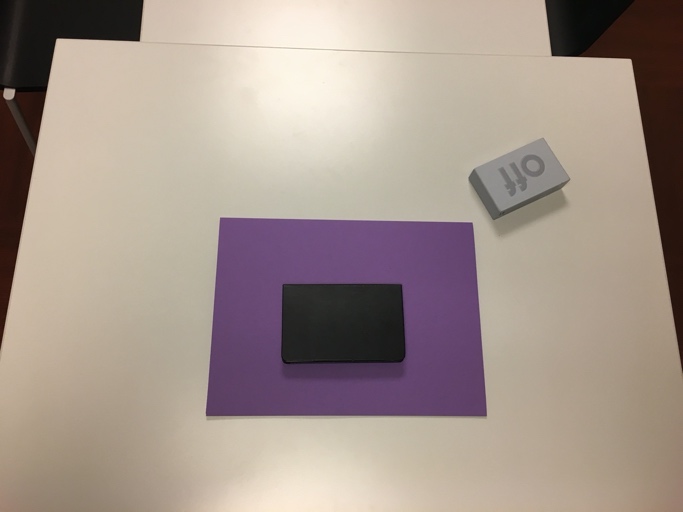
**

1. Smartphone versus notebook (Study 2)


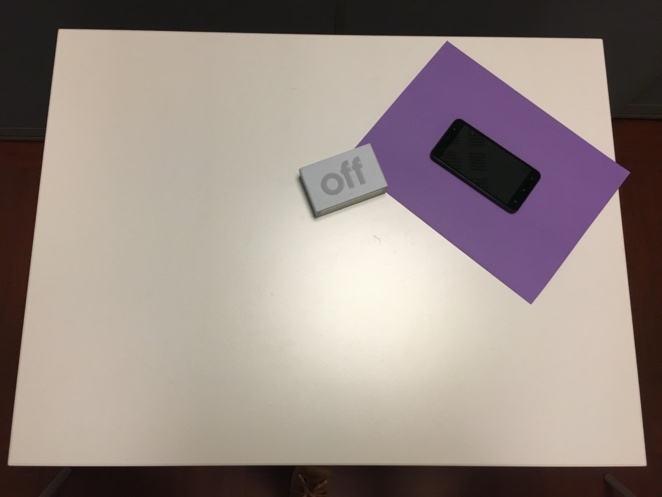

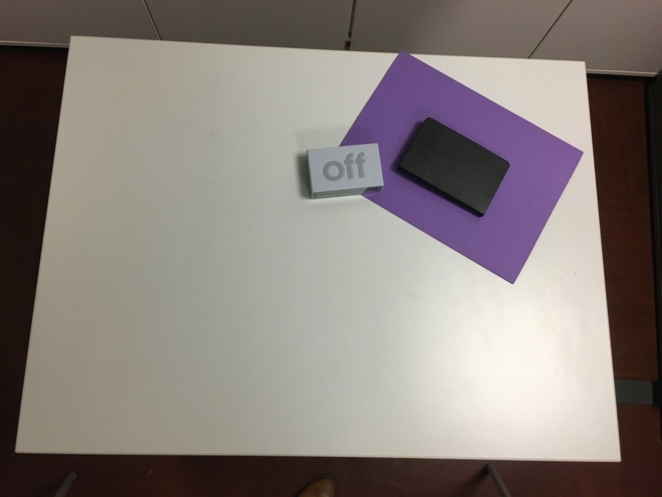

Supplement: S1 Picture — (DOCX) [file pone.0251451.s001.docx]
