# Supplementary material for: How bad is the mere presence of a phone? A replication of Przybylski and Weinstein (2013) and an extension to creativity
Source: PLoS One. 2021 Jun 9;16(6):e0251451. doi: 10.1371/journal.pone.0251451 (PMC8189469; doi:10.1371/journal.pone.0251451)
Supplement: S1 Appendix — (DOCX) [file pone.0251451.s002.docx]

**S1 Appendix. Additional information regarding the manipulation and participants’ reactions to the presence of the smartphone and the notebook (Studies 1 and 2).**

In both studies, participants were exposed to the mere presence of a smartphone or a notebook by random assignment. As in Przybylski and Weinstein (2013), no cover story was provided as to the presence of the objects in order to avoid drawing attention to them and to focus on the effect of the smartphone’s mere presence only. This setting is similar to real life situations in which someone’s smartphone is resting on a table during a conversation or teamwork, without people necessarily knowing whose it is. This seems even more plausible when the group members are strangers to each other.

During the tasks, only a few participants reacted spontaneously to the presence of the objects. In Study 1, as participants were moving to individual cubicles to answer the final questionnaire, two participants from two different groups asked whether the phone belonged to the experimenter or someone else from the lab. One other participant in the notebook condition asked where it came from, that is whether it was placed on the table during the tasks. In Study 2, one participant in the notebook condition and two participants in the smartphone condition asked whether they were being recorded, without directly referring to the objects. When we asked participants what they thought the purpose of the study was at the end of the final questionnaires, no one mentioned the smartphone or the notebook.

In both studies, we included in the questionnaire exploratory measures to investigate whether the participants were aware of the presence of the smartphone (Studies 1-2) and the notebook (Study 2) and if they noticed them, what their thoughts were. In addition, just before ending the sessions, we probed participants orally regarding the presence of the smartphone or notebook. We report these exploratory results for both studies separately.

**Study 1**

In Study 1, we included several exploratory questions in the questionnaire regarding the presence of a phone during the tasks. We asked every participant the same questions, even if they were in the notebook condition. Specifically, we first asked them whether there was a phone on the table during the tasks (Yes / No / Not sure). Out of the 79 participants in the smartphone condition, 56 said there was one (71%), 19 said there wasn’t any (24%) and four were not sure (5%). Among the 35 groups in the smartphone condition, there were two groups in which no participant saw the phone, 16 groups in which part of the group saw it, and 17 groups in which all the participants reported seeing it. We then asked participants who had seen a phone whether they had touched it. Only four said they did. No one in the notebook condition reported seeing a phone.

Exploratory analyses showed that the overall pattern of results from Study 1 held when excluding participants who did not see the phone in the phone condition, when excluding groups where no one saw the phone, or when excluding groups where some participants had not seen the phone. There were only minor changes with some marginal results on isolated items. We detail the differences hereafter.

On the replication measures pertaining to relationship quality, the pattern of results for dyads was the same as with all participants. On triads, it was the same with the exception of a marginal effect of the presence of the smartphone on participants’ feeling of closeness toward their partners (*p* = 0.09), such that triad participants in the notebook condition felt closer to their partners than participants from the smartphone condition. On dyads and triads taken together, the only difference was a marginal difference in the appreciation of the experience (*p* = 0.10), such that participants in the notebook condition appreciated it more.

Regarding the creativity scores, the pattern of results when excluding in the smartphone condition participants or groups that did not see the phone is the same as when including all participants.

The results remained also unchanged on RAT performance, that is there was no effect of the mere presence of the smartphone versus notebook on RAT scores or number of RAT answers, whether for dyads, triads, or dyads and triads taken together. On the creativity process measures from the questionnaire, the overall pattern of results was the same when removing participants or groups that did not see the phone with only minor differences on some measures. One difference was the effect of the smartphone on feelings of autonomy that became significant instead of marginal in the main analysis (participants in presence of the smartphone felt more autonomous). In dyads, there was also a marginal effect of the presence of the smartphone on feelings of motivation when removing participants that did not see the phone and when removing participants from groups in which at least part of the group did not see the phone (*p*s = 0.08), such that participants in the smartphone condition felt more motivated than participants in the notebook condition. There were also marginal effects of the presence of the smartphone on some items for triads. Specifically, when removing participants who did not see the phone, there was a marginal effect of the smartphone on feelings of group cohesion (*p* = 0.07) such that participants in the notebook condition felt more cohesion. When excluding participants from triads in which the entire group did not see the phone, participants in presence of the notebook were marginally more satisfied about their toys than those in presence of the smartphone (*p* = 0.10). When taking only triads in which all the members saw the phone, there was a marginal effect of the smartphone on enjoyment of the task, such that participants in the notebook enjoyed the creative task more (*p* = 0.09).

The oral probe at the end of the session gave us some qualitative information about the thoughts of participants regarding the mere presence of the smartphone and the notebook. Among the 60 participants in the smartphone condition who noticed the phone or thought there might be a phone, 11 did not have any particular thoughts about it, 17 thought someone had forgotten it (a participant, someone from the lab, or the experimenter), 13 thought it might be recording the discussion, 11 thought it would maybe be used later in the session, three just wondered why it was there, two thought it might be a timer, and three had other thoughts. In the oral discussion, we also asked participants in the notebook condition if they had noticed the notebook and their thoughts. Forty-six participants believed they saw it, among which 20 had no particular thoughts, 12 thought it would be used later on, seven thought someone had forgotten it, two thought it might have a microphone hidden in it, two wanted to open it, and three had other thoughts.

**Study 2**

In Study 2, we included in the questionnaire more exploratory questions regarding the presence of the phone or the notebook depending on the condition participants were in. In the smartphone condition, 64 participants reported seeing the phone (61%), 39 not seeing it (37%), and two were unsure (2%). In the notebook condition, 46 participants reported seeing it (44%), 38 not seeing it (36%), and 21 were unsure (20%). Therefore, the smartphone seemed to be more noticeable than the notebook. We also asked participants who saw the objects when they saw them. Participants seemed to have noticed the phone sooner (55% saw it at the beginning of the tasks, 25% around the middle, 16% towards the end, and 4% didn’t remember) than those who saw the notebook in the notebook condition (39% at the beginning, 33% in the middle, 15% at the end, 13% didn’t remember). We then asked participants the extent to which they were surprised by the presence of the object on a 7-point scale from 1 = not at all to 7 = extremely. There was no difference in surprise (*p* = 0.27) between those who noticed the phone in the smartphone condition and those who noticed the notebook in the notebook condition. We asked participants what their thoughts were regarding the object if they were surprised about it. The type of thoughts were similar to those in Study 1. Specifically, among the 54 participants who reported their thoughts, there were 24 thoughts that it might be recording the interactions, 18 that someone had forgotten it, six just wondered why it was there, four whether it would be used later on in the session and there were eight different thoughts (some participants reported several thoughts). We included a question on whether the participants had discussed the objects with their partners during the tasks. Only three participants out of 105 in the notebook condition reported having talked to their partners about the notebook and 12 about the phone in the smartphone condition, with similar topics as the thoughts reported previously. Finally, eight participants said they touched or wanted to touch the phone while 12 said the same thing for the notebook. The oral debriefing at the end revealed similar patterns of answers.

We conducted exploratory analyses to assess the impact of the mere presence of the smartphone on relationship quality, creativity and the creative process when removing participants who did not see the phone (we kept participants who did not see the notebook as it was our control condition), when removing participants in groups where no one saw the phone, and when removing groups in which at least some of the members did not see the phone. Once again, the results on relationship quality were not replicated. There was still no effect of the smartphone on toy creativity. In addition, the analysis on process measures only revealed additional marginal results. When removing the groups where at least part of the members did not see the phone, there was a marginal effect on risk-taking (*p* = 0.08), such that participants in the phone condition felt they took more risk. There was also a marginal effect on approach motivation as measured in the BAS fun seeking subscale (*p* = 0.09), such that participants in the notebook condition had a higher score on this subscale than participants in the smartphone condition.
