# Supplementary material for: How bad is the mere presence of a phone? A replication of Przybylski and Weinstein (2013) and an extension to creativity
Source: PLoS One. 2021 Jun 9;16(6):e0251451. doi: 10.1371/journal.pone.0251451 (PMC8189469; doi:10.1371/journal.pone.0251451)
Supplement: S2 Appendix — Translation from local language. (DOCX) [file pone.0251451.s003.docx]

**S2 Appendix. Instructions for the toy creativity task (Studies 1 and 2).** Translation from local language.

**New product study**

As a group, please draw a new toy, as creative as possible, that a child from 5 to 11 years old can play with.

Your toy can only be composed of the shapes that are given for this study.

You have 15 minutes for this activity.

**Shapes**


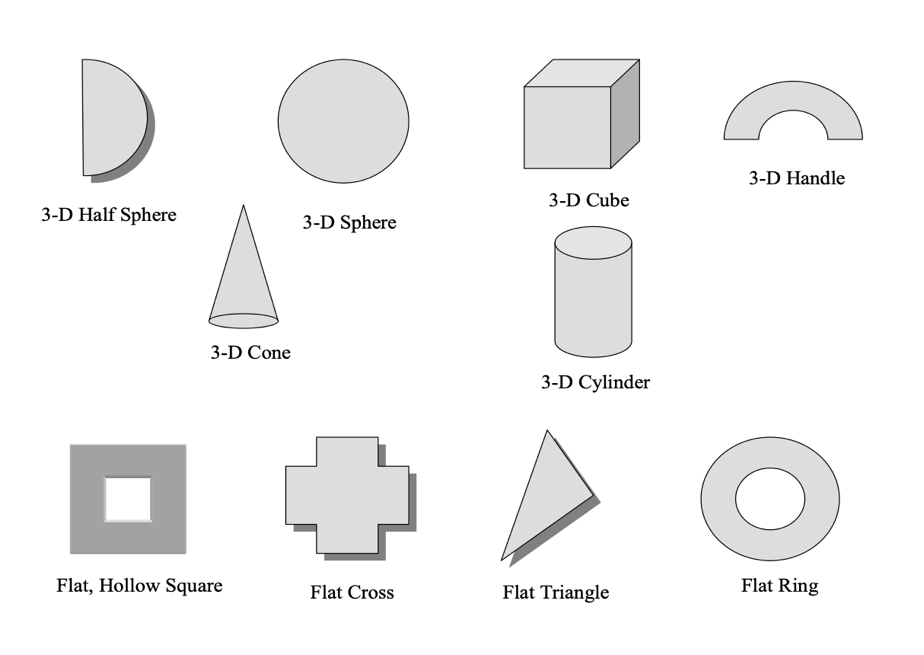


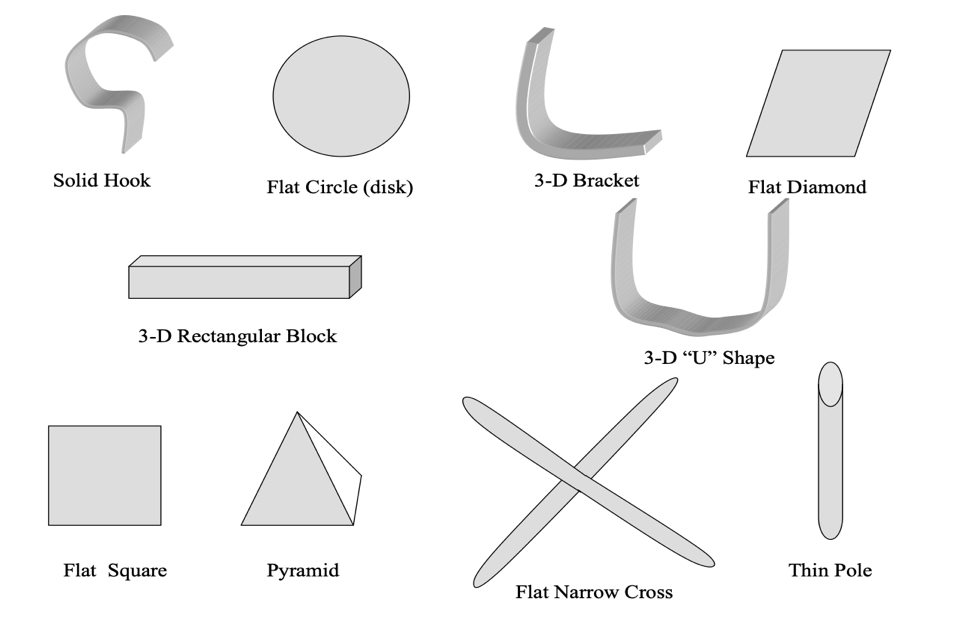


**Reasons why the toy is creative**

Please explain up to 5 aspects of your toy that – according to you - make it creative.
