# Supplementary material for: How bad is the mere presence of a phone? A replication of Przybylski and Weinstein (2013) and an extension to creativity
Source: PLoS One. 2021 Jun 9;16(6):e0251451. doi: 10.1371/journal.pone.0251451 (PMC8189469; doi:10.1371/journal.pone.0251451)
Supplement: S3 Appendix — Translation from local language. (DOCX) [file pone.0251451.s004.docx]

**S3 Appendix. Remote associates test (Study 1).** Translation from local language.

**Word associations**

In this study, you will be provided with either 3 words that are somehow related to another unreported word. Your task is to identify and report the word that you think is related to all other mentioned words.

Here are a couple of examples:

**1. Blank, White, Lines**

One target word in this case would be "Page", as it is related to each of the three words listed above.

**2. Water, Seine, Flow**

One target word in this case would be "River" as it is related to each of the three words listed above.

Your task in this study is to solve up to 10 of these problems in 10 minutes. We are interested in you**trying to come up with one solution when working on a problem.**

Below are the 10 sets of words. Please work on the problems in the order they are presented. Once you figure out a solution, please report it in the space provided and move on to the next problem.

Once you have moved to another problem, please do not go back to previous problems. If you feel that you can't solve the problem, just leave the space blank.

Your solution

Time, Hair, Stretching *Answer: long*

Art, Round, Tennis *Answer: table**

Ache, Hunter, Cabbage *Answer: head**

White, Mouse, “Bleu” *Answer: cheese*

Hit, Nose, Ground *Answer: foot**

Bench, Tube, Literary *Answer: essay**

King, England, Crown *Answer: monarchy*

Nurse, Sick, Occupation *Answer: doctor*

Web, Insect, Creepy *Answer: spider*

Bed, Rest, Pillow *Answer: sleep*

Note. *Some solutions only make sense in the local language.
