# Supplementary material for: How bad is the mere presence of a phone? A replication of Przybylski and Weinstein (2013) and an extension to creativity
Source: PLoS One. 2021 Jun 9;16(6):e0251451. doi: 10.1371/journal.pone.0251451 (PMC8189469; doi:10.1371/journal.pone.0251451)
Supplement: S4 Appendix — Translation from local language. (DOCX) [file pone.0251451.s005.docx]

**S4 Appendix. Final questionnaire (Study 1).** Translation from local language.

1. **Toy creation - Self-assessment and process measures**

Self-assessment of creativity

To what extent do you think your toy corresponds to the following descriptions?

- 1 = Not creative at all to 7 = Extremely creative
- 1 = Not original at all to 7 = Extremely original
- 1 = Not novel at all to 7 = Extremely novel
- 1 = Not useful at all to 7 = Extremely useful
- 1 = Not practical at all to 7 = Extremely practical
- 1 = Not appropriate at all to 7 = Extremely appropriate

Satisfaction

Please rate the extent to which you agree with each statement regarding the toy you designed.

(1 = Strongly disagree to 7 = Strongly agree)

- I like the toy we created
- I am very satisfied with the toy we created
- The toy is a good reflection of my group’s creativity

Process evaluation

How would you describe the process through which you went to create your toy?

- 1 = It wasn’t pleasant at all to 7 = It was very pleasant
- 1 = I didn’t like it at all to 7 = I liked it a lot
- 1 = It was very difficult to 7 = It was very easy
- 1 = It took a lot of time to 7 = It was very quick
- 1 = It was very boring to 7 = It was a lot of fun

Group work appreciation

Please rate the extent to which you agree with each statement regarding your participation to this task in group.

(1 = Strongly disagree to 7 = Strongly agree)

Cohesion

- Our group worked very well
- There was a strong cohesion between the members of our group
- I felt close to the other participant(s) of the group
- The group was very supportive

Group work

- We all put a lot of effort in this activity
- During the creation of the toy, the ties between group members became stronger
- We created a real group spirit on top of creating a toy
- Around the end of the creative exercise, the group shared a series of implicit norms about how each member had to behave
- If we had to work again together as a group, we would perform even better because this task helped us build a group identity

Risk-taking

While your group was creating the toy, to what extent did it take risks in creative efforts?

- 1 = We made conservative choices to 7 = We made bold choices
- 1 = We played it safe to 7 = We took a lot of risks

Intrinsic Motivation Inventory

Please rate the extent to which you agree with each statement about how you felt as you were imagining the toy with your group.

(1 = Strongly disagree to 7 = Strongly agree)

- I put much effort in this activity
- It was important to me to do well in this activity

Competence/autonomy

Please rate the extent to which the following statements correspond to what you felt while you were imagining the toy with your group.

(1 = Strongly disagree to 7 = Strongly agree)

- I felt competent
- I felt free to express myself

Control

To what extent do you agree with the following statements regarding the way you felt while creating the toy?

(1 = Strongly disagree to 7 = Strongly agree)

- I was under the impression I had power
- I felt like the other participant(s) made all the decisions

Concentration

- During the creation of the toy, it was easy for me to concentrate on what I was doing

1. **Relationship measures**

Relationship quality

Please rate the extent to which the following statements correspond to what you felt toward the other participant(s) of your group

(1 = Strongly disagree to 7 = Strongly agree)

*Przybylski and Weinstein (2013):*

- I feel very distant from this person/these people
- I doubt I will ever be friends with the person/people I did the study with
- I felt I could really trust the other participant(s) of the group (trust item #1)
- I would like to have a chance to interact with the other person/people of my group in the future
- I didn’t feel I could trust this person/these people (trust item #2)
- It is possible that this person/these people and me could be friends if we talked more
- I feel close to this person/these people

*Relationship quality (additional items):*

- I felt like I could connect with the other participant(s)
- I felt disconnected from the other participant(s)
- I felt in line with the other participant(s)

Partner closeness (Aron, Aron, and Smollan 1992)

Please select a picture below to describe the extent to which you felt close to the other participant(s) of your group.


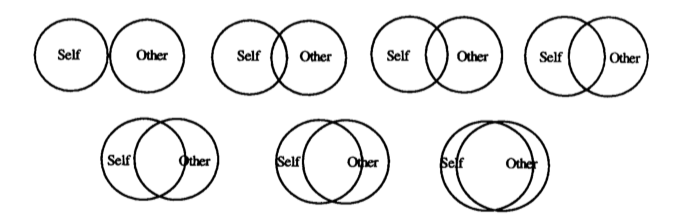


Empathy

*Own empathy:*

To what extent did you feel empathy toward the other member(s) of your group?

- 1 – Not at all
- 2 – A little
- 3 – Moderately
- 4 – Quite a bit
- 5 – Extremely

|  |
| --- |
|  |

*Partner empathy:*

To what extent do you think your partner(s) understood your thoughts and feelings during the group conversation?

- 1 – Not at all
- 2 – A little
- 3 – Moderately
- 4 – Quite a bit
- 5 – Extremely

Interest / Pleasure / Distraction

Please evaluate the way you felt during the group conversation.

(1 = Strongly disagree to 7 = Strongly agree)

- I enjoyed this study a lot
- I would describe this experience as very interesting
- During the study, I found it easy to concentrate
- During the study, I was easily distracted

PANAS

This part of the questionnaire contains adjectives describing feelings and emotions.

For each of these adjectives, please select how you felt during all the group interactions, that is during the group discussion and the toy creation, and not right at this moment.

To do so, please use one of these answers:

- 1 – Not at all
- 2 – A little
- 3 – Moderately
- 4 – Quite a bit
- 5 – Extremely
- Involved
- Anxious
- Excited
- Angry
- Strong
- Guilty
- Scared
- Hostile
- Enthusiastic
- Proud
- Irritated
- Alert
- Ashamed
- Inspired
- Nervous
- Determined
- Attentive
- Agitated
- Active
- Afraid

1. **Smartphone information measures**

How often do you look at your phone?

- Less than once a day
- Once a day
- Several times a day
- Every hour
- Every 30 minutes
- Every 10 minutes
- Every 5 minutes or less

On average, how many hours a day do you spend on your phone?

On average, how many messages a day do you send from your phone?

How much money in euros would you be willing to be paid to spend one entire day without your phone?

Please rate the extent to which you agree on the following statements regarding the use of your mobile phone.

(1 = Strongly disagree to 7 = Strongly agree)

- I check my phone before getting out of bed in the morning
- When I am waiting for a friend, I pass the time by looking at my phone
- If my phone rings or vibrates during a meeting, I look to see what it is about
- I use my phone while driving

Please rate the extent to which you agree on the following statements regarding your relationship to your mobile phone.

(1 = Strongly disagree to 7 = Strongly agree)

- I would have difficulty spending an entire day without my phone
- I feel like I would not be able to live without my phone
- If I forgot to take my mobile phone, I feel nervous
- It drives me mad when my mobile phone is out of battery
- I am upset when I notice I don’t have network on my phone
- I get impatient when my internet connection on my mobile is slow
- I feel lonely when my phone doesn’t ring or vibrate for several hours
- Using my phone makes me happy
- I find I have difficulty concentrating when my phone is not far
- I become less attentive to what surrounds me when I am using my mobile phone
- I would feel lonely if I didn’t have a phone
- I would feel disconnected from my friends if I didn’t have any phone
- I would prefer losing my wallet than my phone

**References**

Aron A, Aron EN, Smollan D. Inclusion of Other in the Self Scale and the structure of interpersonal closeness. J Pers Soc Psychol. 1992 Oct;63(4): 596-612.

Przybylski AK, Weinstein N. Can you connect with me now? How the presence of mobile communication technology influences face-to-face conversation quality. J Soc Pers Relat. 2013 May;30(3): 237-246.
