# Supplementary material for: How bad is the mere presence of a phone? A replication of Przybylski and Weinstein (2013) and an extension to creativity
Source: PLoS One. 2021 Jun 9;16(6):e0251451. doi: 10.1371/journal.pone.0251451 (PMC8189469; doi:10.1371/journal.pone.0251451)
Supplement: S5 Appendix — Translation from local language. (DOCX) [file pone.0251451.s006.docx]

**S5 Appendix. Final questionnaire (Study 2).** Translation from local language.

1. **Toy creation - Self-assessment and process measures**

Self-assessment of creativity

To what extent do you think your toy corresponds to the following descriptions?

- 1 = Not creative at all to 7 = Extremely creative
- 1 = Not novel at all to 7 = Extremely novel
- 1 = Not original at all to 7 = Extremely original
- 1 = Not innovative at all to 7 = Extremely innovative
- 1 = Not useful at all to 7 = Extremely useful
- 1 = Not appropriate at all to 7 = Extremely appropriate

Satisfaction

Please rate the extent to which you agree with each statement regarding the toy you designed.

(1 = Strongly disagree to 7 = Strongly agree)

- I like the toy we created
- I am very satisfied with the toy we created

Mind wandering (inspired from Matthews et al. 1999)

Please rate the extent to which you agree with each statement regarding what happened during the toy creation.

Never, Once, A few times, Often, Very often

- I thought about something that happened earlier in the day
- I thought about something that happened recently
- My mind wandered a great deal
- My thoughts were confused and difficult to control
- I was sometimes not paying attention
- I thought of something that happened long ago
- I thought about something that could happen in the future
- I had too much to think about to be able to concentrate on the task
- I found it hard to maintain my concentration for more than a short time
- I thought of people I know

Process evaluation

How would you describe the process through which you went to create your toy?

- 1 = It wasn’t pleasant at all to 7 = It was very pleasant
- 1 = I didn’t like it at all to 7 = I liked it a lot
- 1 = It was very difficult to 7 = It was very easy
- 1 = It took a lot of time to 7 = It was very quick
- 1 = It was very boring to 7 = It was a lot of fun

Risk-taking

While your group was creating the toy, to what extent did it take risks in creative efforts?

- 1 = We made conservative choices to 7 = We made bold choices
- 1 = We played it safe to 7 = We took a lot of risks

Group work appreciation

Please rate the extent to which you agree with each statement regarding your participation to this task in group.

(1 = Strongly disagree to 7 = Strongly agree)

- Our group worked very well
- There was a strong cohesion between the members of our group
- I felt close to the other participants of the group
- We all put a lot of effort in this activity
- During the creation of the toy, the ties between group members became stronger
- We created a real group spirit on top of creating a toy

Intrinsic Motivation Inventory

Please rate the extent to which you agree with each statement about how you felt as you were imagining the toy with your group.

(1 = Strongly disagree to 7 = Strongly agree)

- I put much effort in this activity
- It was important to me to do well in this activity

Competence/autonomy

Please rate the extent to which the following statements correspond to what you felt while you were imagining the toy with your group.

(1 = Strongly disagree to 7 = Strongly agree)

- I felt competent
- I felt free to express myself

Busyness

To what extent did you feel busy while you were creating the toy with your group?

(1 = Not busy at all to 7 = Extremely busy)

To what extent would you say your thought process was controlled?

(1 = Not controlled at all to 7 = Extremely controlled)

Approach avoidance (Carver and White 1994; Mehta, Dahl, and Zhu 2017 )

To what extent do you agree with the following statements regarding what you felt during the toy creation?

(Totally true, Mostly true, Mostly false, Totally false)

I felt capable of going out of my way

If I did well on this task I'll be willing to do more such studies

I was willing to try something new because I thought it was fun

The idea of creating an original toy made me feel excited and energized

I cared about the fact that my ideas should not draw criticism from people

I was willing to go all out to create an original toy

Because there was a chance to create a truly original toy I moved on it strongly

I was careful enough to not create a toy that may upset or make someone angry

When I saw the opportunity of creating an original toy I got excited right away

I thought the task toy could be beneficial to me and it had an influence on me

I was a bit worried that I'll do poorly at the task

I craved excitement and new sensations

I would say I used a "no holds barred" policy

I worried about making mistakes

1. **Relationship measures**

Relationship quality

Please rate the extent to which the following statements correspond to what you felt toward the other participant(s) of your group

(1 = Strongly disagree to 7 = Strongly agree)

*Przybylski and Weinstein (2013):*

- I feel very distant from this person/these people
- I doubt I will ever be friends with the person/people I did the study with
- I felt I could really trust the other participant(s) of the group (trust item #1)
- I would like to have a chance to interact with the other person/people of my group in the future
- I didn’t feel I could trust this person/these people (trust item #2)
- It is possible that this person/these people and me could be friends if we talked more
- I feel close to this person/these people

*Relationship quality (additional items):*

- I felt like I could connect with the other participant(s)
- I felt disconnected from the other participant(s)
- I felt in line with the other participant(s)

Interest / Pleasure / Distraction

Please evaluate the way you felt during the group conversation.

(1 = Strongly disagree to 7 = Strongly agree)

- I enjoyed this study a lot
- I would describe this experience as very interesting

PANAS

This part of the questionnaire contains adjectives describing feelings and emotions.

For each of these adjectives, please select how you felt during all the group interactions, that is during the group discussion and the toy creation, and not right at this moment.

To do so, please use one of these answers:

- 1 – Not at all
- 2 – A little
- 3 – Moderately
- 4 – Quite a bit
- 5 – Extremely
- Involved
- Anxious
- Excited
- Angry
- Strong
- Guilty
- Scared
- Hostile
- Enthusiastic
- Proud
- Irritated
- Alert
- Ashamed
- Inspired
- Nervous
- Determined
- Attentive
- Agitated
- Active
- Afraid

1. **Smartphone information measures**

How often do you look at your phone?

- Less than once a day
- Once a day
- Several times a day
- Every hour
- Every 30 minutes
- Every 10 minutes
- Every 5 minutes or less

Please rate the extent to which you agree on the following statements regarding your relationship to your mobile phone.

(1= Strongly disagree to 7 = Strongly agree)

- I feel like I would not be able to live without my phone
- If I forgot to take my mobile phone, I feel nervous
- It drives me mad when my mobile phone is out of battery
- I am upset when I notice I don’t have network on my phone
- I feel lonely when my phone doesn’t ring or vibrate for several hours
- I find I have difficulty concentrating when my phone is not far
- I become less attentive to what surrounds me when I am using my mobile phone
- I would feel disconnected from my friends if I didn’t have any phone
- I would prefer losing my wallet than my phone
- When I wait for a friend, I pass time by looking at my phone

**References**

Aron A, Aron EN, Smollan D. Inclusion of Other in the Self Scale and the structure of interpersonal closeness. J Pers Soc Psychol. 1992 Oct;63(4): 596-612.

Carver CS, White TL. Behavioral inhibition, behavioral activation, and affective responses to impending reward and punishment: the BIS/BAS scales. J Pers Soc Psychol. 1994 Aug;67(2): 319-333.

Matthews G, Joyner L, Gilliland K, Campbell S, Falconer S, Huggins J. Validation of a comprehensive stress state questionnaire: Towards a state big three. Personality psychology in Europe. 1999;7: 335-350.

Mehta R, Dahl DW, Zhu RJ. Social-recognition versus financial incentives? Exploring the effects of creativity-contingent external rewards on creative performance. J Consum Res. 2017 Oct 1;44(3): 536-553.

Przybylski AK, Weinstein N. Can you connect with me now? How the presence of mobile communication technology influences face-to-face conversation quality. J Soc Pers Relat. 2013 May;30(3): 237-246.
